# RHAMNETIN IS A BETTER INHIBITOR OF SARS-COV-2 2’-O-METHYLTRANSFERASE THAN DOLUTEGRAVIR: A COMPUTATIONAL PREDICTION

**DOI:** 10.21010/Ajid.v16i2.9

**Published:** 2022-05-06

**Authors:** Adekunle B. Rowaiye, Angus Nnamdi Oli, Olukemi A. Onuh, Nkoli W Emeter, Doofan Bur, Oluwaseun A. Obideyi, Oluyomi Cornelius Dayisi, Jude N. Akpa, Lovelyn Birah, Edward E. Omaka¹, Frances Otibhor Iseghohi¹, Akinbobola Peace Otitoju, Philip F. Uzor, Jude Nnaemeka Okoyeh

**Affiliations:** 1 Department of Medical Biotechnology, National Biotechnology Development Agency, Lugbe, Abuja, Nigeria; 2 Department of Pharmaceutical Microbiology and Biotechnology, Faculty of Pharmaceutical Sciences, Nnamdi Azikiwe University Awka, Nigeria; 3 Department of Pharmaceutical and Medicinal Chemistry, Faculty of Pharmaceutical Sciences, University of Nigeria, Nsukka, Enugu State, Nigeria; 4 Department of Biology and Clinical Laboratory Science, Division of Arts and Sciences, Neumann University, One Neumann Drive, Aston, PA 19014-1298, USA

**Keywords:** SARS-CoV-2, COVID-19, Coronavirus disease, 2’-O-methyltransferase Inhibition, Computational Drug prediction

## Abstract

**Background:**

The 2’-O-methyltransferase is responsible for the capping of SARS-CoV-2 mRNA and consequently the evasion of the host’s immune system. This study aims at identifying prospective natural inhibitors of the active site of SARS-CoV-2 2’O-methyltransferase (2’-OMT) through an in silico approach.

**Materials and methods:**

The target was docked against a library of natural compounds obtained from edible African plants using PyRx - virtual screening software. The antiviral agent, Dolutegravir which has a binding affinity score of -8.5 kcal mol^−1^ with the SARS-CoV-2 2’-OMT was used as a standard. Compounds were screened for bioavailability through the SWISSADME web server using their molecular descriptors. Screenings for pharmacokinetic properties and bioactivity were performed with PKCSM and Molinspiration web servers respectively. The PLIP and Fpocket webservers were used for the binding site analyses. The Galaxy webserver was used for simulating the time-resolved motions of the apo and holo forms of the target while the MDWeb web server was used for the analyses of the trajectory data.

**Results:**

The Root-Mean-Square-Deviation (RMSD) induced by Rhamnetin is 1.656A^0^ compared to Dolutegravir (1.579A^0^). The average B-factor induced by Rhamnetin is 113.75 while for Dolutegravir is 78.87; the Root-Mean-Square-Fluctuation (RMSF) for Rhamnetin is 0.75 and for Dolutegravir is 0.67. Also, at the active site, Rhamnetin also has a binding affinity score of -9.5 kcal mol^−1^ and forms 7 hydrogen bonds compared to Dolutegravir which has -8.5 kcal mol^−1^ and forms 4 hydrogen bonds respectively.

**Conclusion:**

Rhamnetin showed better inhibitory activity at the target’s active site than Dolutegravir.

## Introduction

In November 2019, in Wuhan China, the index case of 2019-nCoV (COVID-19), an infectious disease whose etiological agent is a new strain of coronavirus (SARS-CoV-2) was identified (Saghazadeh and Rezaei, 2020).

By January 2020, the World Health Organization pronounced the disease a public health crisis and in March, 2020, a pandemic due to the ravaging effect and global exponential spread (Arshad Ali et al., 2020). COVID-19 has been known to spread from animals and the human-to-human mode of transmission has been established through contact with respiratory droplets and body fluids from infected persons. The symptoms of this disease include fever, loss of taste, loss of smell, cough and difficult breathing (Rowaiye *et al.*, 2020). In critical cases, the complications include pneumonia, acute respiratory distress syndrome, failure of multiple organs and death (Topcuoglu, 2020).

Taxonomically SARS-CoV-2 is classified as a beta-coronavirus, a genus which comprises of Middle East respiratory syndrome (MERS-CoV), the severe acute respiratory syndrome (SARS-CoV), Bat SARS-related coronaviruses, (SARSr-CoV) and other species found in humans and animals (Zhao *et al.*, 2020). SARS-CoV-2 contains an approximately 30 kbp genome and this consists of genes that code for 29 different proteins that include structural, non-structural and accessory proteins (Jin *et al.*, 2020). The structural proteins provide structural support and facilitate attachment, entry into host cell, assembly and pathogenesis. They include the envelope, membrane, nucleocapsid and spike proteins (Satarker and Nampoothiri, 2020).

pp1a and pp1ab are the two long polypeptides of the SARS-CoV-2 which are divided into smaller 16 non-structural proteins (NSP1-NSP16) (Satarker and Nampoothiri, 2020). The non-structural proteins consist of enzymes such as protease, methyltransferase, helicase, primase, polymerase and endoribonuclease which are involved in various physiological and pathological processes. The processes include replication and recombination (Ogando et al., 2020), translation and mitochondrial biogenesis (Gvozdjakova *et al.*, 2020), the inhibition of type I IFN expression (Thoms *et al.*, 2020), inhibition of host antiviral replication mechanisms (Cascella *et al.*, 2020) and evasion of host’s immune surveillance (Gvozdjakova *et al.*, 2020).

The SARS-CoV-2 has 9 accessory proteins which also facilitate infectivity, virulence, ion channel formation, virus release (Issa *et al.*, 2020), inhibition of IFN-1, the glycosylation of the interferon-inducible transmembrane protein, Tetherin and other processes involved in viral metabolism and host pathology (Konno *et al.*, 2020). The discovery and development of effective antiviral agents against the SARS-CoV-2 would serve as an important strategy in combating the pandemic. Dolutegravir has been shown to be an inhibitor of the active site of 2′-OMT (Khan *et al.*, 2020).

This study evaluates the activities of an enzyme; 2’ O-methyl transferase (2′-OMT) which engages in an important role in 2′O-methylation of the guanine-N7 methylated capped RNA that allows the virus to circumvent the immune system of the host. This enzyme is an important drug target as its inhibitors would make the virus more visible to host immune attack.

## Materials and methods

### Analysis and validation of the structure of target

From the I-TASSER webserver, the three-dimensional structure of SARS-CoV-2 2′-OMT (ID: QHD43415_15. pdb) was downloaded. It has a predicted Template Modelling (TM) score of 0.99 (Roy *et al.*, 2010). The architecture of SARS-CoV-2 2′-OMT was obtained by using the VADAR (version 1.8) web server. Using the MolProbity web server, further analysis and validation of the target’s structure was performed with the Ramanchandran plot (Chen *et al.*, 2017). The PyMol software and Proteinplus web server were used for the visualization of the target protein (DeLano, 2002; Fährrolfes *et al.*, 2017).

### Ligand preparation

The 3D structures of 1,048 natural compounds found in African fruits, vegetables, and spices were obtained from the PubChem database in their structure-data file (SDF) format. The 3D structure of the standard, Dolutegravir (PubChem CID 54726191) was also obtained from PubChem (Kim *et al.*, 2019). The compounds were earlier screened for compliance with Veber [number of rotatable bonds ≤ 10, topological polar surface area (TPSA) ≤ 140), and Lipinski [hydrogen bond donor (HBD) ≤ 5, hydrogen bond acceptor (HBA) ≤ 10, molecular weight ≤ 500 and Lipophilicity-Index (logP) ≤ 5] rules (Veber *et al.*, 2002; Lipinski, 2004).

### Molecular docking and virtual screening

Through the Open Babel plug-in tool on the Virtual Screening Molecular Docking Software, PyRx (version 0.8), all the ligands were uploaded. To prepare for molecular docking, the 3D structures of the natural compounds were converted from the SDF format to the “Protein Data Bank, Partial Charge & Atom Type (PDBQT) format” (Dallakyan and Olson, 2015). The Universal Force Field (UFF) was set as the energy minimization parameter while the conjugate gradient descent was used as the optimization algorithm. The standard and all the ligands were docked against SARS-CoV-2 2-OMT using the AutoDock Vina plug-in tool in PyRx with the following grid parameters: Centre X = 61.0369, Y = 60.9705, Z = 61.1676; and Dimensions (Angstrom): X = 59.5445, Y = 45.8966, Z = 54.6730 (Dallakyan and Olson, 2015). The results of the dockings were downloaded in the comma-separated values (CSV) format for further screening. The reference score was -8.5 kcal mol^−1^ which is the binding affinity between Dolutegravir and the target. The SWISSADME web server was used for the prediction of the molar refractivity (Daina *et al.*, 2017). The pkCSM web server was used to perform the pharmacokinetic properties’ prediction (Pires *et al.*, 2015). Bioactivity prediction of the ligands against six different classes of drug targets was performed using the Molinspiration webserver (Molinspiration, 2020).

Binding site analyses of the complexes formed by the target protein and ligands was performed using the PyMol software and the PLIP web server. The analyses include hydrogen bonds, hydrophobic interactions, and salt bridges (DeLano, 2002; Salentin *et al.*, 2015). The Fpocket web server was used to characterize the binding pockets domiciled in the target (Le Guilloux *et al.*, 2009).

### Molecular Dynamic Simulations (MDS) and Analyses

The GROMACS plug in tool of the Galaxy supercomputing server was used to execute a 2-nanosecond MDS of the apo- and holoproteins (Afgan *et al.*, 2018). The parameterization of the ligands was performed using LigParGen server. Topology files that are compatible with GROMACS were generated for the ligands with force field parameter set at OPLS-AA/ 1.14*CM1A (Afgan *et al.*, 2018). On the Galaxy platform, the PDB files of the apo and holo forms of the target were converted to topology files. Thereafter, solvation (energy minimization to reduce steric clash), equilibration (NVT and NPT) and a one million step MDS was performed. On the Galaxy server, the BIO 3D tool was used for the analyses of the trajectories (Afgan *et al.*, 2018). The analyses include Principal Component Analysis (PCA), Root Mean Square Deviation (RMSD), Dynamical Cross-Correlation Map (DCCM), Root Mean Square Fluctuation (RMSF) and Hydrogen bond analysis (Dodda *et al.*, 2017). The MDWeb web server was used to perform the radius of gyration and B-factor predictions (Abraham *et al.*, 2015).

## Results

### Structural analysis and validation of the target

**Figure 1:**
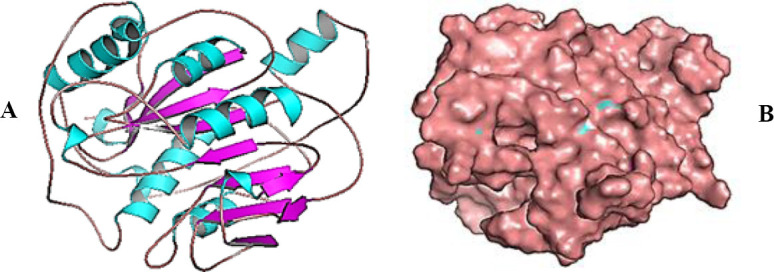
(A) the crystal structure of SARS-CoV-2 2′-OMT (cartoon model): Beta sheets in magentas, Alpha helix in cyan, and Loops in pink. (B) Surface representation of the target

**Figure 2:**
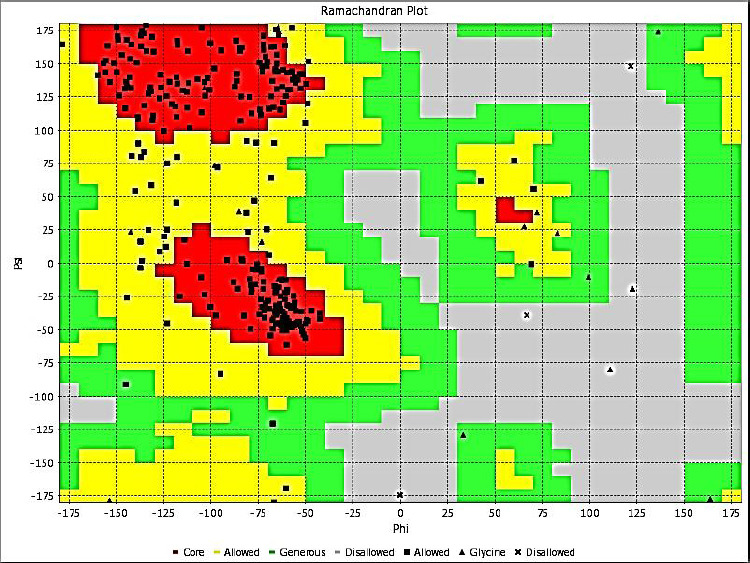
Ramanchandran diagram of the target

The apo structure of the target has 298 amino acid residues with the secondary structures as follows: alpha helix 23%; beta sheets 30%; coil 45%; and turns 18% (Fig. 1). The total solvent-accessible surface area (SASA) is 13,990.4(Å). The geometry of the target shows 6.20% poor rotamers, 88.37% favored rotamers, 1.01% Ramanchandran outliers, 87.84% Ramanchandran favored, 1.80% Carbon Beta deviations >0.25Å, 0.00% bad bonds and 0.49% bad angles (Fig. 2). The Peptide omegas of the target include 0.34% Twisted Peptides and 0.00% Cis Prolines. The low-resolution criteria include 4.8% C-Alpha Based Low-resolution Annotation Method (CaBLAM) outliers, and 0.34% C-Alpha Geometry outliers.

### Molecular descriptors of ligands

**Figure 3:**
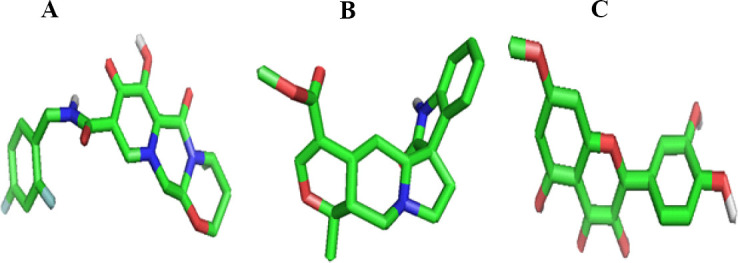
The 3-dimensional structures of the standard and leads (stick model) (A) Dolutegravir (B) Isopteropodin (C) Rhamnetin

(Fig. 3 and Table 1): The molecular descriptors such as HBA, HBD, log P, molecular weight and TPSA for the standard and lead compounds does not exceed 10, 5, 5, 500 g/mol, and 140 angstroms respectively. Also, the molar refractivity for the standard and lead compounds ranged between 40 and 130, while their number of rotatable bonds does not exceed 10.

**Table 1:** Molecular descriptors of standard and leads

Variables	Dolutegravir (Standard)	Isopteropodin	Rhamnetin
**Molecular Weight (g/mol)**	419.38	368.4	316.26
**XLogP3**	2.40	1.6	1.9
**Hydrogen Bond Donors**	2	1	4
**Hydrogen Bond Acceptors**	8	5	7
**Number of non-hydrogen atoms**	30	27	23
**Number of bonds that can rotate**	3	2	2
**Topological PSA (A^a^)**	99.2	67.9	116
**Molar Refractivity**	104.48	106.47	82.5
**Ligand of GPCR**	0.05	0.37	- 0.11
**Modulator of Ion channels**	-0.20	0.25	- 0.27
**Inhibitor of Kinases**	-0.04	- 0.34	0.21
**Ligands of Nuclear Receptors**	-0.20	0.07	0.27
**Inhibitor of Proteases**	0.04	- 0.02	- 0.27
**Inhibitor of Enzymes**	0.07	- 0.02	0.2

For bioactivity, the enzyme inhibition prediction values for Dolutegravir and Rhamnetin are greater than 0.00 while that of Isopteropodin is less than 0.00. No compound shows any bioactivity prediction value less than -5.00 (Table 1)

### Pharmacokinetic properties of ligands

From Table 2, the leads and the standard have water solubility values greater than -4.0 log mol/L**.** The Caco2 permeability (log Papp in 10^-6^ cms^-1^) values for the standard and Isopteropodin are greater than 0.9 while the value for Rhamnetin is less than 0.9. The human intestinal absorption (% absorbed) values for all the compounds are greater than 30. For skin permeability (LogKp), all the compounds have values less than −2.5 (Table 2).

**Table 2:** ADMET properties of ligands

Variables	Dolutegravir (Standard)	Isopteropodin	Rhamnetin
**Solubility in water (log mol/L)**	-3.139	-3.521	-3.212
**Permeability in caco2 cells (log Papp in 10^-6^ cm.s^-1^)**	1.133	1.119	-0.361
**Human Intestinal absorption (%)**	74.36	96.483	80.214
**Dermal Permeability (log Kp)**	-2.839	-3.767	-2.735
**Substrate of P-gp (Yes/No)**	Yes	Yes	Yes
**Inhibitor of P-gp I (Yes/No)**	No	No	No
**Inhibitor of P-gp II (Yes/No)**	No	No	No
**Volume of Distr. Steady State (human) (log L/kg)**	-0.069	0.845	0.419
**Fraction unbound (human)**	0.169	0.357	0.073
**Permeability of Blood-Brain Barrier (log BB)**	-1.02	0.035	-1.345
**Permeability of CNS (log PS)**	-3.614	-2.307	-3.235
**Substrate of CYP2D6 (Yes/No)**	No	No	No
**Substrate of CYP3A4 (Yes/No)**	Yes	Yes	No
**Inhibitor of CYP1A2 (Yes/No)**	No	No	Yes
**Inhibitor of CYP2C19 (Yes/No)**	No	No	No
**Inhibitor of CYP2C9 (Yes/No)**	No	No	No
**Inhibitor of CYP2D6 (Yes/No)**	No	No	No
**Inhibitor of CYP3A4 (Yes/No)**	No	No	No
**Total Clearance (log ml/min/kg)**	-0.062	0.886	0.473
**Substrate of Renal OCT2 (Yes/No)**	No	Yes	No
**AMES toxicity (Yes/No)**	No	No	No
**Max. Tolerated dose (human) (log mg/kg/day)**	0.035	-1.088	0.56
**Inhibitor of hERG I (Yes/No)**	No	No	No
**Inhibitor of hERG II (Yes/No)**	No	No	No
**Oral Rat Acute Toxicity (LD50) (mol/kg)**	1.921	2.763	2.453
**Oral Rat Chronic Toxicity (log mg/kg/day)**	1.393	1.771	2.679
**Hepatotoxicity (Yes/No)**	Yes	Yes	No
**Dermal Sensitization (Yes/No)**	No	No	No
***T. Pyriformis* toxicity (log µg/L)**	0.301	0.526	0.331
**Minnow toxicity (log mM)**	3.1	-0.364	1.885

For distribution pharmacokinetics, the fraction unbound values for the leads and the standard are greater than 0.1. The BBB permeability (log BB) values for Dolutegravir and Rhamnetin are less than -1.0 while that of Isopteropodin is greater than -1.0 but less than 0.3. The values for CNS permeability (Log PS) for Rhamnetin and the standard are less than-3.0, while the value for Isopteropodin is greater than -3.0 but less than -2.0. The Volume of distribution steady state (Log VDss) value for Isopteropodin is greater than 0.45; but greater than 0.15 and less than 0.45 in Rhamnetin and Dolutegravir. The leads and the standard are all substrates of P-glycoprotein and non-inhibitors of P-glycoprotein I & II. Similarly, all the compounds are non-substrates of CYP2D6 and are also not inhibitors of CYP3A4, CYP2C19, CYP2D6, and CYP2C9 enzymes. Only Rhamnetin is predicted to be a CYP1A2 inhibitor and a non-substrate of CYP3A4 enzymes (Table 2).

Isopteropodin has the highest Total Clearance (log ml/min/kg) value while Dolutegravir has the least. Also, only Isopteropodin is a substrate of Renal OCT2. All the compounds showed no AMES toxicity, no skin sensitization, and non-inhibition of hERG I & II proteins. Only Rhamnetin is predicted to be non-hepatotoxic. The values for maximum tolerated dose (log mg/kg/day) and Oral Rat Chronic Toxicity (log mg/kg/day) is highest in Rhamnetin and the value for Oral Rat Acute Toxicity (LD50) (mol/kg) is highest in Isopteropodin. The values of *T. Pyriformis* toxicity (log µg/L) for lead compounds and standard are all greater than -0.5. Only Isopteropodin has a Minnow toxicity (log mM) value less than 0.3 (Table 2)

Analysis of molecular docking of ligands against the target shows that Rhamnetin exhibited the lowest binding score (with the target protein) of -9.5 Kcal/mol, followed by Isopteropodin with a score of -8.6 Kcal/mol and then Dolutegravir (Standard) with a score of -8.5 Kcal/mol

### Binding Site analyses

**Figure 4:**
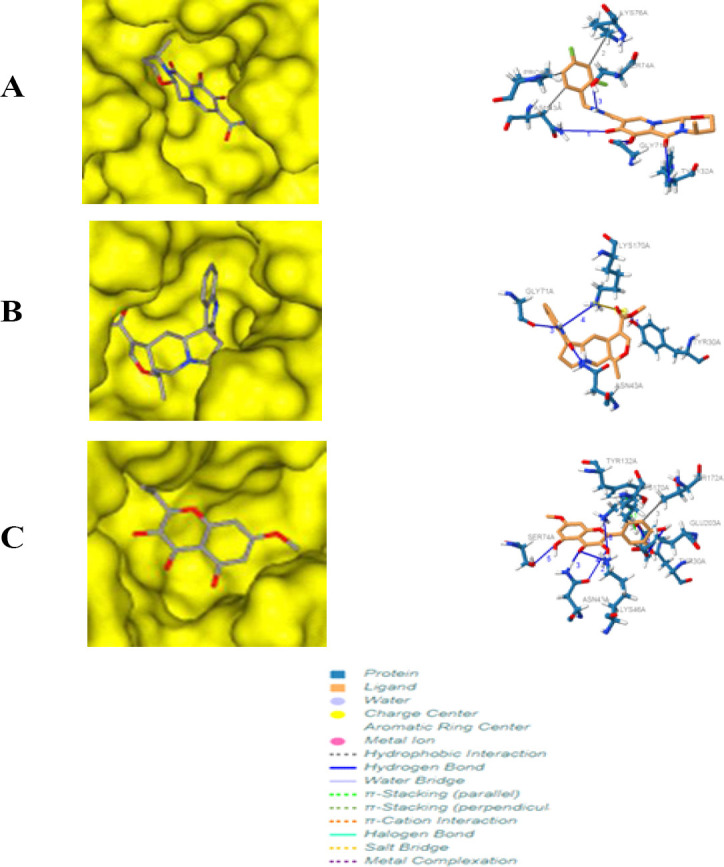
Binding site of the target interacting with the standard and leads. (a) 2’-OMT-Dolutegravir complex, (b) 2’-OMT-Isopteropodin complex (c) 2’-OMT-Rhamnetin complex

The Pocket 21 of SARS-CoV-2 2-OMT consists of the following residues: GLU203, ASN198, THR172, LYS170, TYR132, ASP130, ASP75, SER74, GLY73, GLY71, LYS46, ASN43, MET42, TYR30, and ASN29 [20]. Fig. 4 and Table 3 reveal that all the hydrogen bonds of the standard and leads fall within Pocket 21. Also, of all the compounds, Rhamnetin had the highest number of hydrogen bonds formed with the target. With respect to the angles formed by the hydrogen bonds, the standard and the target form four hydrogen bonds with angles less than 130° and no bond angle greater than 130°. Isopteropodin only forms one hydrogen bond at LYS170 while Rhamnetin forms only one hydrogen bond at ASN43. Remarkably, all three compounds form a hydrogen bond at ASN43. With regards to the donor to acceptor distance, the standard forms no hydrogen bond within the range of 2.5-3.2 Å and four hydrogen bonds within the range of 3.2-4.0 Å with the target (Table 3A). Isopteropodin forms one hydrogen bond within the range of 2.5-3.2 Å and three hydrogen bonds within the range of 3.2-4.0 Å. Rhamnetin forms two hydrogen bonds at ASN43 and SER74 within the range of 2.5-3.2 Å and five hydrogen bonds within the range of 3.2-4.0Å.

**Table 3:** Analysis of Hydrogen bonds

A	Complexes	No. of bonds	Residues	Distance (H-A)	Distance (D-A)	Bond angle	
	2’-OMT- Dolutegravir	4	ASN43	3.33	3.82	111.15	
			GLY71	2.35	3.02	125.93	
			SER74	2.96	3.66	125.37	
			TYR132	2.66	3.4	129.64	
	2’-OMT- Isopteropodin	4	TYR30	2.41	2.97	116.73	
			ASN43	2.97	3.33	101.14	
			GLY71	2.74	3.42	123.96	
			LYS170	3.19	3.98	135.16	
	2’-OMT- Rhamnetin	7	TYR30	3.51	3.84	102.71	
			ASN43	3.12	3.81	130.06	
			ASN43	2.78	3.15	101.72	
			LYS46	3.23	3.92	126.29	
			SER74	2.35	2.85	111.48	
			LYS170	2.64	3.28	121.36	
			GLU203	3.27	3.86	122.83	
**B**	**Complexes**	**Hydrophobic Interaction**		**Salt bridge**		**p-Stacking**	
		**Residue**	**Distance**	**Residue**	**Distance**	**Residue**	**Distance**
	2’-OMT- Isopteropodin			LYS170	4.19		
	2’-OMT- Dolutegravir	ASN43	3.4				
		LYS76	3.62				
		PRO80	3.58				
	2’-OMT- Rhamnetin	TYR30	3.98			TYR132	4.27
		LYS170	3.8				
		THR172	3.31				

From Table 3B, 2’-OMT-Dolutegravir and 2’-OMT-Rhamnetin complexes both have three hydrophobic interacts each. The 2’-OMT- Isopteropodin and 2’-OMT-Rhamnetin complexes each have a salt bridge and a p-stacking respectively.

From Table 3B, 2’-OMT-Dolutegravir and 2’-OMT-Rhamnetin complexes both have three hydrophobic interacts each. The 2’-OMT- Isopteropodin and 2’-OMT-Rhamnetin complexes each have a salt bridge and a p-stacking respectively.

### Root Mean Square Deviation (RMSD)

Supplementary File_Fig 1 and Table 4 reveal the RMSD of apo and holo forms of the target predicted over 20 timeframes (100 picoseconds each) in a 2-nanosecond trajectory. The 2’OMT-Isopteropodin complex had the highest average and total RMSD values of all the holo forms of the target. The 2’-OMT-Rhamnetin complex also had higher average and total RMSD values than the 2’OMT-Dolutegravir complex. Similarly, the trajectories plateaued on time frames 13, 19, and 16 for the Dolutegravir, Isopteropodin, and Rhamnetin complexes respectively. The RMSD distribution values of the 2’-OMT-Isopteropodin and 2’-OMT-Rhamnetin complexes all fall between 1.0 – 2.49 Å while that of the 2’-OMT-Dolutegravir complex falls between 1.0 – 1.99 Å. Four peaks of the 2’-OMT-Isopteropodin complexes fall within the 2.00 – 2.49 Å range.

**Table 4:** MDS of the apo and holo forms of the target (a summary)

MDS Parameters	2’-OMT-Apo	2’-OMT -Dolutegravir	2’-OMT - Isopteropodine	2’-OMT -Rhamnetin
**RMSD**				
**Total RMSD**	36.716	33.169	37.545	34.782
**Average RMSD**	1.748	1.579	1.788	1.656
**Lowest RMSD**	0	0	0	0
**Highest RMSD**	2.328	1.96	2172	2.047
**Time frame of highest RMSD**	21	13	19	16
**Time frame of lowest RMSD**	1	1	1	1
**RMSD Peak Distribution**				
**0.00-0.49A**	1	1	1	1
**0.50-0.99A**	0	0	0	0
**1.00-1.49A**	4	6	1	1
**1.50-1.99A**	7	14	15	17
**2.00-2.49A**	9	0	4	2
**2.50-2.99A**	0	0	0	0
**3.00-3.49A**	0	0	0	0
**RMSF**				
**Total Global RMSF**	260.75	250.96	236.79	251.62
**Average Global RMSF**	0.87	0.84	0.79	0.84
**Total Regional (Pocket 21) RMSF**	9.83	10.01	10.31	12.16
**Average Regional (Pocket 21) RMSF**	0.66	0.67	0.69	0.75
**Least Fluctuation**	0.33	0.30	0.29	0.34
**Highest Fluctuation**	6.21	4.93	5.47	5.31
**Range of RMSF**	5.88	4.62	5.18	4.97
**PCA**				
**Total global motions (mean of PC1, PC2 & PC3)**	12.14	12.92	11.71	12.4
**Average global motions (mean of PC1, PC2 & PC3)**	0.04	0.04	0.04	0.04
**Total Regional (Pocket 21) Motion (mean of PC1, PC2 & PC3)**	0.39	0.52	0.45	0.59
**Average Regional (Pocket 21) Motion (mean of PC1, PC2 & PC3)**	0.02	0.03	0.03	0.04
**Best global Conformation**	PC3	PC2	PC2	PC3
**Best regional Conformation (Pocket 21)**	PC2	PC3	PC2	PC1
**PC1 Eigen Value**	44.10%	36.31%	33.66%	29.49%
**PC2 Eigen Value**	10.91%	12.69%	16.90%	17.00%
**PC3 Eigen Value**	7.55%	8.84%	10.15%	10.70%
**Total**	62.56%	57.84%	60.71%	57.19
**PC1 cosine content**	0.81	0.6	0.85	0.86
**PC2 cosine content**	0.15	0.12	0.75	0.37
**PC3 cosine content**	0.29	0	0.09	0.27
**B-Factor**				
**Global Average B-Factor**	121.79	109.77	107.41	133.92
**Regional (Pocket 21) Average B-Factor**	94.36	78.87	98.59	113.75
**Radius of Gyration**				
**Average Gyration**	3.5926	3.5863	3.5865	3.5922
**Minimum Gyration**	3.59856	3.59305	3.59251	3.60286
**Maximum Gyration**	3.58136	3.57493	3.58255	3.58148
**Range Gyration**	0.0172	0.0181	0.00996	0.02138
**% Gyration**	0.48	0.5	0.28	0.6
**Time Frame of Maximum Gyration**	16	19	19	20
**Time Frame of Minimum Gyration**	1	1	16	1

### Root mean square fluctuation (RMSF)

The 2’-OMT-Rhamnetin complex had the highest total and average global values and is followed closely by the 2’-OMT-Dolutegravir which has same average global value with 2’-OMT-Rhamnetin complex. The least is the 2’-OMT-Isopteropodin complex. For the regional (Pocket 21) fluctuation, the 2’-OMT-Rhamnetin complex had the highest total and average values followed by the 2’-OMT-Isopteropodin complex and then the 2’-OMT-Dolutegravir complex (Supplementary File_Fig. 2 and Table 4). Specifically, on ASN43 which is a residue that all the compounds bind on within the active site, 2’-OMT-Rhamnetin complex showed the greatest fluctuation of the holo structures (0.71). At this residue the 2’-OMT-Isopteropodin complex (0.68) also showed greater than 2’-OMT-Dolutegravir complex (0.59).

### Principal Component Analysis (PCA)

From Supplementary File_Fig. 3 and Table 4, the cumulative of the first three highest principal components (PC1, PC2, and PC3) for all the holo forms of the target are responsible for more than 50% of the total variance. The total global motion (the average of PC1, PC2 and PC3) was highest in the 2’-OMT-Dolutegravir complex and least in the 2’-OMT-Isopteropodin complex. However, the total regional motion (the average of PC1, PC2, & PC3) was highest in 2’-OMT-Rhamnetin complex followed by the 2’-OMT-Dolutegravir complex. Based on the greatest motion, the best global conformations are PC3 for the 2’-OMT-Rhamnetin complex and PC2 for 2’-OMT-Dolutegravir and 2’-OMT-Isopteropodin complexes. Similarly, the conformations that produced the greatest motions at Pocket 21 are PC2 of the 2’-OMT-Isopteropodin complex, PC3 for the 2’-OMT-Dolutegravir complex and PC1 for the 2’-OMT-Rhamnetin complex. Of all the holo forms of the target, the 2’-OMT-Rhamnetin complex has the highest motion at the regional level (Pocket 21) while the 2’-OMT-Isopteropodin complex has the least. The PCA cosine content of the dominant motions related to PC1 for all the holo forms of the target did not get to 1.0.

### Radius of Gyration (RoG)

From the graphical plots, the 2’-OMT-Rhamnetin complex shows a steep upward slope, indicating that it is the least compact of all the complexes (Supplementary File_Fig. 4). Data from Table 4 suggests that over the trajectory, the 2’-OMT-Rhamnetin complex has the highest values for the range of gyration and the percentage gyration. This is followed by the 2’-OMT-Dolutegravir complex.

### The dynamic cross-correlation analysis

From Supplementary File_Fig. 5, the most intense overall anti-correlated motion of amino acid residues occurred mainly in the 2’-OMT-Rhamnetin complex between residues 1-150 which is within the active site of SARS-CoV-2 2’-OMT. This complex also showed correlated motions between residues 70-100. The 2’-OMT-Dolutegravir and 2’-OMT-Isopteropodin complexes showed mainly non-correlated motions between amino acid residues 1-200.

### B-factor

The 2’-OMT-Rhamnetin complex has the highest global and regional B-Factor values than other holo forms of the target. While the 2’-OMT- Isopteropodin complex has a lower B factor value than the 2’-OMT- Dolutegravir complexes at the global level, it has a higher value at the regional (Pocket 21) level (Supplementary File_Fig. 6 and Table 4).

## Discussion

### Molecular descriptors of ligands

The bioavailability of a compound largely determines its absorption and permeation into desired cellular targets. As described by Lipinski, Ghose, and Veber rules, several key factors determine bioavailability. These molecular descriptors include molecular weight ≤ 500 g/mol, log P ≤ 5, HBD ≤ 5, HBA ≤ 10, molar refractivity between 40 and 130, TPSA ≤ 140 and the number of rotatable bonds ≤ 10 (Veber *et al.*, 2002; Lipinski, 2004; Hospital *et al.*, 2012). The standard and leads did not violate the Lipinski, Ghose, and Veber rules. These results suggest good oral bioavailability and that all the compounds would be good drug candidates. However, the molecular weights of the lead compounds suggest they would permeate faster into cell membranes than the standard.

Beyond strong binding with a desired target, a drug candidate should elicit the desired bioactivity. Computational drug discovery measures the activity of ligands against the six major drug targets which are nuclear receptors, G-Protein-Coupled Receptors, Kinases, Ion channels, proteases, and other enzymes. Bioactivity is evaluated to determine the efficacy of the drug and its mechanism of action. Bioactivity scores greater than 0.00 is considered active; −5.0 and 0.0 are moderately active; and less than -5.0 is inactive (Al Wasidi *et al.*, 2020). As an enzyme inhibitor, Rhamnetin showed the greatest bioactivity. The standard showed greater enzyme inhibiting activity than Isopteropodin.

### ADMET properties of ligands

To avoid pitfalls in the drug discovery process, drug candidates must possess good absorption, distribution, metabolism, excretion, and toxicity profile (*Pires et al.*, 2015). Absorption is the first stage of pharmacokinetics, and it facilitates the movement of a drug from the route of administration to the bloodstream. The process of absorption involves passive diffusion, receptor-mediated endocytosis and active transport. Good absorption parameters include water solubility greater than -4.0 Log mol/L, human intestinal absorption greater than 30%, Caco-2 permeability higher than 0.9, and skin permeability lower than −2.5 (LogKp). The results show that the leads and the standard are water soluble and have high permeability for skin, intestine, and human epithelial colorectal adenocarcinoma (Caco-2).

Distribution is the reversible movement of drug between the different compartments inside the body. This is aided by certain physicochemical properties of the drug which helps it to permeate the biological membranes into the tissues, and organs of the body. Distribution can be evaluated by pharmacokinetics markers such as Fraction unbound (greater than 0.1), Volume of distribution steady state (High: Log VDss > 0.45; Low: Log VDss <- 0.15), CNS permeability (permeable Log PS > -2; poor Log PS < -3), and BBB permeability (permeable: Log BBB > 0.3; poor <: Log BBB <-1) (Pires *et al.*, 2015). The standard showed low VDSS, Isopteropodin has high VDss, but the VDss value of Rhamnetin falls within pharmacological range. Most of Dolutegravir is predicted to remain in plasma with low levels in tissue. More of Isopteropodin and Rhamnetin are predicted to be available in the tissues. However, Rhamnetin shows the best distribution balance between plasma and tissue. The fraction of unbound for Rhamnetin is 0.073 suggesting that 92.7% of the drug is bound to plasma reducing drug concentration at the site of action and reducing the rate of elimination (Smith *et al.*, 2015). The standard and Rhamnetin have a poor BBB and CNS permeability suggesting that only Isopteropodin would effectively reach the brain, and CNS (Pires *et al.*, 2015). P-glycoprotein (P-gp) is a plasma membrane transporter protein which actively pumps its substrates outside the cell through an ATP-dependent efflux mechanism. The standard and the two leads are predicted to be P-gp substrates which suggest that they should be co-administered with a P-gp inhibitor to increase their utilization. On the contrary, all compounds are non-inhibitors of P-gp I and II suggesting that they would not alter the function of the P-gp efflux pump.

Drug metabolism prediction seeks to investigate the metabolic activity of a particular candidate with respect to the major isomers of the Cytochrome-P450 enzyme responsible for their biotransformation. Drug candidates are substrates, inhibitors, or inducers of these enzymes (Issa *et al.*, 2017). Only Rhamnetin is an inhibitor of CYP1A2 suggesting that it should not be administered with substrates of this enzyme as it would prevent their metabolism resulting in toxic accumulation. In a similar vein, the standard and Isopteropodin are substrates of CYP3A4 suggesting that they should not be administered with the inhibitors of that enzyme.

To avoid toxic accumulation, drug excretion is extremely important. The total clearance is the sum of all body clearances which includes renal, hepatic, and pulmonary. Renal clearance is the most common which is physiologically considered as the rate of excretion divided by the plasma concentration of drug. Drug plasma concentration is affected by the initial dose administered and the half-life. The predicted values for total clearance show that the standard is most slowly excreted. The excretion rate of Isopteropodin is more than two times higher than that of Rhamnetin. Isopteropodin is also predicted to be a substrate of Renal OCT2 suggesting that it will be eliminated into the urine from the proximal tubules (Li *et al.*, 2016). Toxicity prediction is a very important part of computational drug discovery. The key toxicity profiles considered were genotoxicity, hepatotoxicity, dermatotoxicity, and cardiotoxicity.

For the maximum tolerated dose, values higher than 0.477 log mg/kg/day are interpreted as a high and values less than 0.477 log mg/kg/day are interpreted as low. Isopteropodin has the lowest maximum recommended tolerated dose suggesting that it is the most potent. Rhamnetin has the highest value showing it is the least potent (Pu *et al.*, 2019).

The values of the predicted oral rat acute and chronic toxicities should be interpreted with factors such as dose, drug concentration, the length of time it is administered, and route of administration (Pu *et al.*, 2019).

Biological species such as *Tetrahymena pyriformis*, a protozoan and flathead Minnows, a fish are used for evaluating drug toxicity. The concentration of a drug that can cause death in 50% of the population is considered toxic. For *T. pyriformis*, pIGC50 values greater than -0.5 log Ug/L and for flathead Minnows, log LC50 values less than 0.3 log mM are considered toxic. Data from Table 2 suggests that the standard and the two leads have an antibacterial effect. However, only Isopteropodin is toxic to fish (Pu *et al.*, 2019). The standard and the two leads are predicted to be non-dermatotoxic, non-genotoxic and non-cardiotoxic. However, the standard and Isopteropodin are predicted to be hepatotoxic and this is dose related.

### Molecular docking analyses of ligands and the target

The ability of small molecules to achieve an optimum structural positional conformation in the binding pocket of a protein is measured by the binding affinity score obtained in molecular docking. The ligands with the lowest binding energy scores have the greatest binding affinities and are usually the possible drug candidates (Lin, 2019). The leads are predicted to have greater potency because they have stronger binding affinities than the standard. Rhamnetin is predicted to be the most potent.

### Binding Site analyses

Hydrogen bond plays an essential role in protein-ligand interactions as it enhances binding by displacing water molecules. Both the direction and specificity of ligand binding is determined by the orientation and the length of an intermolecular hydrogen bond (Nounou, 2014). In molecular simulations, an increased number of H-bonds between protein and ligand is suggestive of a stronger binding affinity (Sanchez, 2019). The bioactivity of the leads and the standard can be effectively compared because their hydrogen bonds fall within Pocket 21. Also, of all the compounds, Rhamnetin had the highest number of intermolecular hydrogen bonds suggesting it has the strongest binding affinity with the target protein. With respect to the angles formed by hydrogen bonds, the standard forms no strong (greater than 130°) and four weak (less than 130°) hydrogen bonds with the SARS-CoV-2 2’OMT. Isopteropodin only forms one strong hydrogen bond at LYS170 while Rhamnetin forms only one strong hydrogen bond at ASN43. Remarkably, all three compounds form a hydrogen bond at ASN43 (Chen *et al.*, 2016).

With respect to the donor to acceptor distance, the standard forms no moderate (2.5-3.2 Å) and four weak (3.2-4.0 Å) hydrogen bonds with the SARS-CoV-2 2’OMT. Isopteropodin forms one moderate and three weak hydrogen bonds. Rhamnetin forms two moderate bonds (at ASN43 and SER74) and five weak bonds (Chen *et al.*, 2016). The presence of salt bridges, hydrophobic interactions, and p-Stacking further strengthens and stabilizes the target-ligand complexes (Chandrasekar *et al.*, 2019).

### Root Mean Square Deviation (RMSD)

The RMSD measures structural stability and conformational changes during simulation or ligand binding. It measures the average distance between the alpha carbon atoms of the protein backbone. RMSD values higher than 1.0 Å show major conformational change and structural instability. Lower RMSD values suggest the opposite (Bell and Zhang, 2019). Looking at the trajectories, the 2’-OMT-Rhamnetin complex forms the steepest slope of all the holo structures suggesting that with more simulation time, the RMSD values would increase. All the holo structures showed major conformational changes as they had most peaks above 1.0Å. 2’OMT-Dolutegravir complex showed the least structural instability. The 2’OMT-Isopteropodin complex showed the greatest instability followed by the 2’-OMT-Rhamnetin complex.

### Root mean square fluctuation (RMSF)

The function of a protein is largely determined by its structure and dynamics. The motion of a protein is evaluated by the motions of the amino acid residues. The RMSF determines the residual fluctuation of apo and holo proteins during a trajectory (Hassan *et al.*, 2018). The results suggest that Rhamnetin induces the highest fluctuation at the active site of 2’-OMT and hence the greatest inhibitory effect. Isopteropodin also induces a greater inhibitory effect than Dolutegravir at the active site.

### Principle Components Analysis (PCA)

The structural conformations of the holo protein generated during molecular dynamics simulation is statistically evaluated with PCA. The principal components generated are the representative structures of the clusters of conformations generated. From the results, the 2’-OMT-Rhamnetin had the greatest motions at the active site. This suggests that Rhamnetin induced the greatest molecular instability at the active site of SARS-CoV-2 2’-OMT. This is followed by Dolutegravir. Due to the short simulation time, the PCA cosine content of the dominant motions related to PC1 for all the holo forms of the target did not get to 1.0. The simulation is not converged and would require more time. Convergence shows accuracy, sampling quality, and reproducibility. The results of cosine content show good quality for all the holo forms of the target (Dodda *et al.*, 2017)

### Radius of Gyration

Radius of Gyration (RoG) is used in determining alteration in protein complex compactness. RoG is a measure of the mass of atoms comparative to the center of mass of the protein complex (Sawle and Ghosh, 2016). A low RoG suggests the tight packing of the protein while a high RoG suggests the opposite (Tou *et al.*, 2013). The Radius of gyration data generated correlates with the graph indicating that Rhamnetin induces the least compactness followed by the standard and lastly, Isopteropodin.

### B-factor

The B-factor also, referred to as temperature factor is a measure of the variability of the positions of the atoms in line with average atomic coordinates. Furthermore, B-factor gives the necessary information concerning the protein dynamics or the level of unpredictability in the mode (Sneha and Doss, 2016). The results suggest that Rhamnetin caused the most temperature-dependent atomic vibrations thereby producing the highest dynamic disorder of the 2’OMT stereochemistry.

### The dynamic cross-correlation (DCC) analysis

Dynamical cross correlation map is a common method for evaluating the trajectories of the MDS. It shows the pattern of atomic correlations in protein dynamics. Fluctuation patterns induced by ligand binding can also be easily evaluated (Kasahara *et al.*, 2014). The greatest anti-correlation motions at active site were found in the 2’-OMT-Rhamnetin complex suggesting the greatest inhibitory activity.

Derived from Quercetin, Rhamnetin is an O-methylated flavonol isolated from Cloves *Syzygium aromaticum)*. The inhibitory activity of Rhamnetin and its derivative, isorhamnetin against Influenza A virus, West Nile virus, and Human rotavirus has been established (Daniels et al., 2017). Specifically, Rhamnetin has also been predicted to be an inhibitor of SARS-CoV-2 main protease (Civra et al., 2017).

## Conclusion

Dolutegravir (the standard), Rhamnetin and Isopteropodin are all predicted to have good oral bioavailability. Of all the compounds, Rhamnetin showed the greatest enzyme inhibition prediction. The standard is predicted to be better than Isopteropodin in this regard. Pharmacokinetically, all the compounds are water soluble but Isopteropodin is remarkably predicted to be toxic to fish. Molecular Docking Simulation suggests that Rhamnetin is the most potent of the three (with binding affinity score = -9.5 Kcal.mol^-1^) and it also forms the highest number of intermolecular hydrogen bonds. At the active site of 2’-OMT (Pocket 21), Rhamnetin is predicted to have the greatest inhibitory activity as revealed by the highest fluctuations (RMSF), the greatest motions (PCA), highest B-Factor value, greatest anti-correlation motions, and the highest range of gyration over the trajectory. Specifically, on residue ASN43 which all the compounds form a hydrogen bond with, only Rhamnetin forms a strong bond and has the highest RMSF value on this residue. Overall Rhamnetin is predicted to be a better inhibitor of SARS-CoV-2 2’-OMT than Dolutegravir while Isopteropodin is not better.

Further investigation of the inhibitory activities of Rhamnetin on the target through *in-vitro* and *in-vivo* tests is recommended. Also, while drug development for COVID-19 is on the way, there is the need to revisit the national policy on disinfection, train and re-train health workers on the policy and its application for improved healthcare (Oli et all 2013).

### Declarations

### Ethics approval and consent to participate:

Not applicable

### Conflict of Interest:

The Authors declare that there is no conflict of Interest associated with this study.

**List of abbreviations:**
2’-OMT: “2’O-methyltransferase”
B factor: “Debye–Waller factor” or “Temperature factor”
BBB: “Blood-Brain Barrier”
Caco-2 cells: “human colorectal adenocarcinoma cells”
CNS: “Central Nervous System”
COVID-19: “2019 novel coronavirus disease”
CYP: “Cytochromes P450 enzyme”
DCCM: “Dynamical Cross-Correlation Matrix”
GPCR: “G-protein-coupled receptors”
hERG: “The human ether-a-go-go related gene”
IFN: “Interferon”
MDS: “Molecular Dynamics Simulation”
mRNA: “messenger ribonucleic acid”
NSP: “Non-Structural Proteins”
PCA: “Principal Component Analysis”
Pdb: “Protein Data Dank”
Pdbqt: “Protein Data Bank, Partial Charge, & Atom Type”
Pgp: “P-glycoprotein”
Pyrx: “Python prescription”
Renal OCT2: “Renal organic cation transporter 2”
RMSD: “Root Mean Square Deviation”
RMSF: “Root Mean Square Fluctuation”
RoG: “Radius of Gyration”
SARS-CoV-2: “Severe acute respiratory syndrome coronavirus 2”
TPSA: “Topological polar surface area”
UFF: “Universal Force Field”
VDss: “Steady state volume of distribution”
